# Sexual and reproductive health of women living on illegal gold mining sites in French Guiana: a multicentric cross-sectional survey

**DOI:** 10.3389/fgwh.2026.1887897

**Published:** 2026-10-26

**Authors:** Maylis Douine, Alice Sanna, Teddy Bardon, Marion Petit-Sinturel, Lorraine Plessis, Carlotta Carboni, Raphaëlle Le Querriou, Josiane Miller, Jane Bordallo-Miller, Aude Lucarelli, Astrid Van Melle, Céline Michaud, Célia Basurko, Mathieu Nacher, Antoine Adenis, Stephen Vreden, Martha Suarez-Mutis, Yann Lambert

**Affiliations:** 1 Centre d’Investigation Clinique Guyane Amazonie, Inserm 1424, Département recherche-Innovation-Santé Publique, Centre Hospitalier Universitaire de Guyane – Site Cayenne, Cayenne, French Guiana; 2 UMR UA17 Santé Des Populations en Amazonie, Cayenne, French Guiana; 3 Laboratory of Parasitic Diseases, Institute Oswaldo Cruz/Fiocruz, Rio de Janeiro, Brazil; 4 DPAC-Fronteira NGO, Oiapoque, Brazil; 5 Coordination Régionale de la Santé sexuelle (Coress), Centre Hospitalier Universitaire de Guyane – Site Cayenne, Cayenne, French Guiana; 6 Centres délocalisés de Prévention et de Soins (CDPS), Centre Hospitalier Universitaire de Guyane – Site Cayenne, Cayenne, French Guiana; 7 Foundation for the Advancement of Scientific Research in Suriname (SWOS), Paramaribo, Suriname

**Keywords:** gender-based violence, hard-to-reach populations, mining, sexual and reproductive health, STI’s, vulnerability

## Abstract

**Background:**

Women involved in illegal gold mining in French Guiana face intersecting vulnerabilities related to migration, informality, and precarious living and working conditions, which heighten their exposure to sexual and gender-based violence and undermine sexual and reproductive health. However, quantitative data on these issues in this population remain scarce. This study aimed to document the sexual and reproductive health of these marginalized women.

**Methods:**

A cross-sectional descriptive survey was conducted among women involved in illegal gold mining. Participants were recruited at logistical bases at the borders with Suriname and Brazil. After providing informed consent, participants were interviewed by trained field investigators using a standardized questionnaire. It collected information on sociodemographic characteristics, exposure to sexual and gender-based violence, engagement in transactional sex, obstetrical history, access to gynecological care, and testing for sexually transmitted infections (STIs). Data were analyzed using descriptive statistical methods.

**Results:**

Among the 115 women included, 14.0% reported sexual assault or rape (95% CI: 8.2–21.8), and 19.1% reported intimate partner violence. Regarding transactional sex, 9.5% of women were currently engaged in sex work, and 8.7% reported past involvement, among whom 80.9% reported systematic condom use. Overall, 15.6% of women reported a history of induced abortion (95% CI: 9.5–23.6), and half were performed by a healthcare provider. Most participants (80.0%) reported up-to-date gynecological follow-up (95% CI: 71.5–86.8). Previous HIV testing was relatively common (65.2%), whereas testing for hepatitis B (33.9%), hepatitis C (28.7%), and syphilis (35.6%) were less frequent.

**Conclusions:**

Women working in mining areas faced high levels of sexual and intimate partner violence, while transactional sex appeared less aligned with commonly held stereotypes. Despite relatively good access to gynecological care, abortion rates remained high and STIs screening coverage could be improved. Promoting systematic screening for gender-based violence within sexual and reproductive health services is essential.

**Clinical Trial Registration:** NCT05540470.

## 1 Background

Sexual and reproductive health (SRH) encompasses a broad range of services and rights, including access to contraception, fertility and infertility care, maternal and perinatal health services, prevention and treatment of sexually transmitted infections (STIs), protection from sexual and gender-based violence, and education on safe and healthy relationships (1).

Violence against women remains one of the most pervasive and under-addressed human rights issues worldwide (2). Nearly one in three women is estimated to have experienced physical or sexual violence by an intimate partner or sexual violence by a non-partner during her lifetime (2). Globally, approximately 263 million women have been subjected to sexual violence by a non-partner since the age of 15, with profound consequences for women's physical, sexual, and mental health.

Migrant women are disproportionately exposed to sexual and gender-based violence. While migration may offer economic or social opportunities, it often involves cumulative risks along the migration trajectory. These risks are heightened in contexts characterized by informality, weak institutional protection, and precarious living conditions.

In the Amazonian region, illegal gold mining represents a specific migration context marked by high mobility, informality, and precarious working and living conditions. In mining camps, regulation does not rely on formal external oversight but rather on internal mechanisms, where social norms and power relations—embedded in work organization and living arrangements—structure behavior and maintain order. Masculinity is “often constructed around virility and physical strength, justified by the physically demanding nature of gold extraction”, reinforcing gender hierarchies and unequal relationships (3).

In French Guiana (FG), the gold mining population is predominantly composed of migrants, mainly from Brazil, with frequent cross-border movements, either directly from Brazil or through Suriname. Women represent approximately 30% of the gold mining population in FG and are primarily engaged in support activities such as cooking or vending (4, 5). According to Mendes et al., women may migrate to mining areas following invitations from partners already working there, in search of economic opportunities and improved living conditions, or as a means of escaping previous situations of domestic or intimate partner violence (IPV) (5). Within mining camps, women must continuously adapt to ensure their survival while navigating informal and often unequal contractual arrangements. They face structural vulnerabilities, including social isolation, limited access to health services, economic dependency, and heightened exposure to sexual and gender-based violence.

These conditions profoundly affect women's sexual and reproductive health and limit their access to contraception, gynecological and obstetrical care, STIs prevention and testing services, contributing to unmet SRH needs. In this context, sexual violence constitutes both a direct violation of women's rights and a key determinant of adverse SRH outcomes. Ensuring access to comprehensive SRH services is therefore a key public health and human rights priority, particularly for women in marginalized contexts.

Despite these vulnerabilities, existing research on women in gold mining settings has largely focused on heavy metal exposure, maternal and child health outcomes, or STIs epidemiology, rather than on sexual violence and broader SRH indicators (4, 6, 7). Although qualitative and legal studies document severe sexual violence in mining areas, robust quantitative data on sexual violence and SRH among women involved in illegal gold mining remain scarce, especially in the Guiana Shield region (8–10). Women living on illegal gold-mining sites in French Guiana thus constitute a hidden, hard-to-reach population for whom little is known about sexual and reproductive health needs and access to care.

This study addressed this gap by assessing sexual and reproductive health among women living on illegal gold mining sites in French Guiana. We examined exposure to sexual violence, engagement in transactional sex, gynecological and obstetrical follow-up, and access to STIs testing, with the aim of informing public health interventions and policies adapted to the realities of women involved in gold mining in the Amazonian region.

## 2 Methods

### 2.1 Study population and sampling size

The study population comprised persons engaged in informal gold mining activities in French Guiana. Eligibility criteria included being present on a mining site within the seven days preceding enrollment, providing written informed consent, and being aged 18 years or older. The present analysis focused on participants who identified as cisgender women.

### 2.2 Study design

This research consisted of a cross-sectional descriptive survey using data collected through a structured questionnaire. It was conducted as part of the interventional Curema project, designed to assess an innovative strategy for malaria elimination in this vulnerable population (11). The required sample size was determined according to the primary objective of the survey, estimating malaria prevalence. Assuming a 2% baseline prevalence and a 75% reduction after two years, 860 participants per survey were required (α = 0.05, power = 80%) (11).

Study participants were identified in logistical rear bases, where gold miners routinely stop to trade gold, purchase supplies, rest, or visit relatives. These bases are situated along the Maroni River, at the border with Suriname, and the Oyapock River, at the border with Brazil. Recruitment was undertaken by a multidisciplinary team composed of a health mediator and a nurse. A physician in the team at the Surinamese border. After checking the eligibility criteria, the written informed consent form was obtained.

The recruitment process combined chance encounters with a snowball sampling approach, which is particularly suited to engaging hidden or hard-to-reach populations. Participant enrollment occurred between December 2024 and April 2025 (12).

### 2.3 Data collection

The questionnaire included variables related to socioeconomic status, gold mining activity and lifestyle. Women were asked specific questions regarding exposure to violence, transactional sex, obstetrical history, gynecological follow-up and STIs testing. In addition, all participants (both women and men) were asked broader questions regarding exposure to sexual, psychological, and physical violence. This allowed assessment of the consistency of responses among female participants. These broader violence-related questions were part of an ancillary study assessing psychosocial vulnerabilities among individuals involved in gold mining (Douine et al., publication in progress).

The questionnaire was adapted from previous surveys conducted among gold miners and from questionnaire used to assess sexual and reproductive health among persons living with HIV or migrants (7, 13, 14). It was pre- tested on five individuals from the target population to assess both comprehension and acceptability of the questions. Some questions related to secondary objectives had to be shortened or clarified to improve understanding and reduce administration time. The questionnaire is presented in Supplementary Material 1.

In the field, data collection procedures differed by study site. At the Surinamese border, questions specific to women were administered by a female community health worker, while questions regarding STIs testing and general exposure to violence were asked by a female medical doctor. At the Brazilian border, the entire questionnaire was administered by a female nurse. All interviews took place in remote settings sheltered by tarpaulins. Participation was voluntary, and participants could agree to take part in the study while choosing not to answer specific questions.

### 2.4 Statistical analysis

Descriptive statistics were used to summarize the data given the small sample size. Categorical variables were presented as frequencies and percentages, and continuous variables as medians with interquartile ranges. Missing data were explicitly reported when existing for each variable and were not imputed. When relevant, comparisons between groups were performed using standard statistical tests: chi-square or Fisher's exact tests for categorical variables, and Student's *t*-test or Mann–Whitney *U* test for continuous variables, depending on distributional assumptions. Proportions were presented with their 95% confidence intervals to aid interpretation of the estimates. All analyses were conducted using Stata version 19 (StataCorp, College Station, TX, USA).

### 2.5 Ethical approval and consent to participate

Written informed consent was obtained from all participants after providing information in their native language regarding the primary objective of the study (malaria) and the secondary objectives (assessment of sexual health and violence among women).

As part of the Curema project, the study received approval from the Surinamese Ministry of Public Health (N°CMWO005) and from the Brazilian National Research Ethics Commission (N°5.507.241). All data were anonymized and registered in compliance with the General Data Protection Regulation (GDPR). The study was carried out in accordance with the principles of the Declaration of Helsinki.

### 2.6 Participant support and referral procedures

Participants who expressed a need for medical, psychosocial, or other support were referred, with their consent, to the nearest appropriate healthcare or support facility. Depending on their location and needs, referrals were made to healthcare centres in French Guiana or to DPAC, an organization specialized in health mediation, including support for gender-based violence, in Oiapoque, Brazil.

In addition, interviewers received regular supervision throughout the data-collection period. This supervision provided an opportunity to discuss the emotional impact of collecting sensitive information and to receive appropriate support when needed.

## 3 Results

### 3.1 Study population profile

During the 5-month study period, 489 individuals were enrolled: 371 men, 117 women, and one participant who identified with another gender identity and was not included in the present study. Two women had missing data because of technical issues with the digital questionnaire; therefore, the analyses were conducted among 115 women. The median age was 42 years (Interquartile range (IQR): 35–48; range: 21–69). Most participants were born in Brazil (106/115) (mainly Maranhão, Pará and Amapá), followed by Venezuela (*n* = 2), the Dominican Republic (*n* = 4), and Suriname (*n* = 3). Table 1 presents the socio-demographics characteristics of the study population. Among them, 72 were cook, 26 traveling vendor, 4 *garimpeira* (working directly on the mine), 3 sex workers (spontaneous declaration), 3 machine operators, 1 boatman, and 4 accompanying their partner (each participant could report more than one activity).

**Table 1:** Characteristics of the study population, Orpal4, French Guiana, 2024–2025 (*N* = 115).

Variable	Category	*n*	%
Age	≤49 years	88	77.4
	>49 years	26	22.6
Country of inclusion	Suriname	88	76.5
	Brazil	27	23.5
Country of birth	Brazil	106	92.2
	Maranhão	44	51.5
	Pará	36	34.0
	Amapá	9	8.5
	Amazonas	4	3.8
	Piaui	4	3.8
	Ceará	4	3.8
	Roraima	2	1.9
	Other brazilian states^a^	3	2.8
	Dominican Republic	4	3.5
	Suriname	3	2.6
	Venezuela	2	1.7
Education level	primary or none	50	43.5
	secondary or higher	65	56.5
Police operation in past year	No	60	52.2
	Yes	55	47.8
Distance to mining site	<1 day	63	56.8
	≥1 day	48	43.2
Financial situation	Comfortable/moderate	76	66.1
	Difficult	23	20.0
	Dnk/dnwta^b^	16	13.9
Time in mining (*N* = 474)	≤10 years	59	51.3
	>10 years	56	48.7

a Parana, Paraïba, Bahia.

b Do not know/do not want to answer.

### 3.2 Sexual and gender-based violence

#### 3.2.1 Rape and sexual assault

Twenty-five women reported knowing someone who had been a victim of rape or sexual assault (21.7%, 95% CI: 14.6–30.4). Thirteen women reported having personally experienced rape or sexual assault (11.3%, 95% CI: 6.2–18.6), including nine aggressions occurring since 2020 and four before 2020. Three women answered “no” to this question but later reported psychological or physical pressure for sexual intercourse or unwanted sexual acts, and were therefore counted as victims of sexual assault. Thus, the proportion of victims was 14.0% (95% CI: 8.2–21.8). Ten women reported being both witnesses and victims.

The majority of reported assaults took place in French Guiana (Figure 1). Given that logistical rear bases and places of residence are typically located in Brazil or Suriname, incidents occurring in French Guiana are most often linked to mining sites.

**Figure 1:**
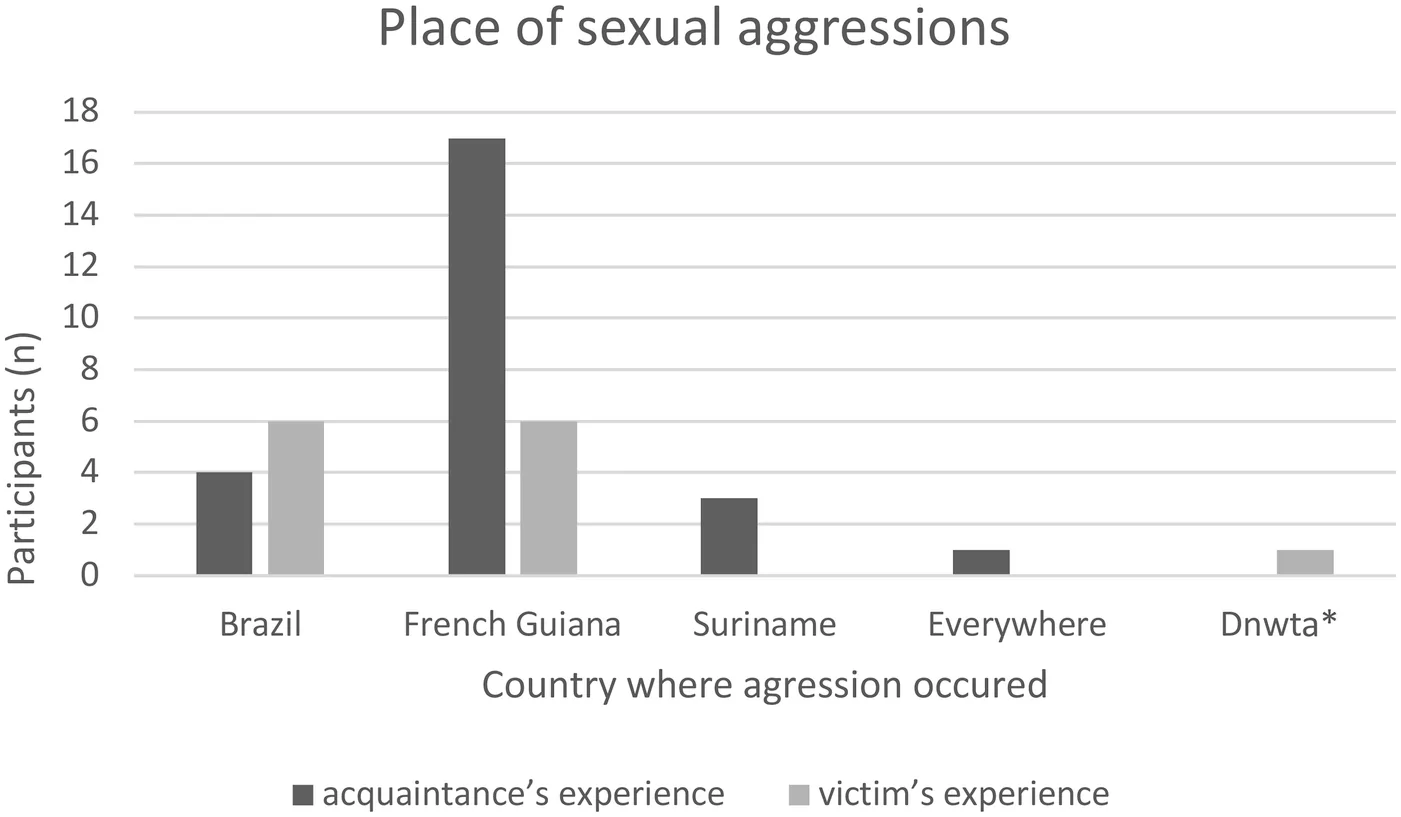
Place of sexual agression of women working in illegal mining in French Guiana, 2024–2025. *Dnwta, Do not want to answer.

Women between 30 and 49 years old were more concerned by sexual violence. No association was found between having experienced sexual assault or rape and country of birth, country of inclusion or education (Table 2).

**Table 2:** Sociodemographic characteristics of female victims of sexual assaut in gold mining areas, French Guiana, 2024–2025.

Variable	Category	Study population		Sexual agression				
		*n*	%	Yes *n* (%)		No *n* (%)		*p-value*
				*n*	%	*n*	%	
Age	<30 years	13	11.3	0	0	13	100	0.037
	31–49 years	76	80.0	15	66.1	60	20.0	
	≤50 years	26	22.6	1	3.8	25	96.2	
Country of inclusion	Suriname	88	76.5	14	16.1	73	83.9	0.212
	Brazil	27	23.5	2	7.4	25	92.6	
Country of birth	Brazil	106	92.2	16	15.1	90	84.9	0.286
	Other	9	7.8	0	0	8	100	
Education level	Primary or none	50	43.5	8	16.0	42	84.0	0.593
	Secondary or higher	65	56.5	8	12.5	56	87.5	
Police operation in past year	No	60	52.2	5	8.3	55	91.7	0.065
	Yes	55	47.8	11	20.4	43	79.6	
Distance to mining site	<1 day	63	56.8	9	14.3	54	85.7	0.818
	≥1 day	48	43.2	6	12.8	41	87.2	
Financial situation	Comfortable/moderate	76	66.1	8	14.3	48	85.7	0.979
	Difficult	23	20.0	3	15.0	17	85.0	
	Dnk/dnwt^a^	16	13.9	5	13.2	33	86.8	
Time in mining (*N* = 474)	≤10 years	59	51.3	6	10.3	52	89.7	0.248
	>10 years	56	48.7	10	17.9	46	82.1	

a Dnk/dnwta, Do not know/do not want to answer.

#### 3.2.2 Intimate partner violence

Twenty-two women reported having experienced IPV (22/115, 19.1%; 95%CI: 12.4–27.5). In half of these cases, IPV occurred in a context in which the partner and/or participant, her partner, or both had consumed alcohol. These partner violences were not associated with the participant's age or with the duration of involvement in gold-mining activities.

### 3.3 Transactional sex work

Twenty-one women reported ever engaging in transactional sex work, and one declined to answer (21/115, 18.3%; 95% CI: 11.7–26.5). Among these 21 women, five engaged in sex work frequently, six occasionally, and ten had done so in the past but were no longer active. They were between 21 and 51 years old. Eleven women were thus engaged in transactional sex work at the time of the study (11/115, 9.5%; 95% CI: 4.9–16.5) and 8.7% in the past (10/115, 8.7%; 4.2–15.4). Among them, ten reported five or fewer partners in the previous year, and one reported more than 20 partners. Sex work was not associated with country of birth.

Among the 21 women who had ever engaged in sex work, 17 (80.9%) reported always using condoms, one frequently, one occasionally, one never, and one responded “don't know”. Four women reported that condom use was sometimes or frequently subject to negotiation with clients.

All women who practiced sex work did so directly at mining sites. Fifteen worked independently (71.4%), two worked for a third party (9.5%), one employed someone for her own security (4.7%), and three declined to answer. Thus, among those who responded, 88.9% (16/18) were working independently. Reported primary occupations included cook (*n* = 8), travelling vendor (*n* = 5), sex worker (*n* = 3), machine owner (*n* = 2), shop employee (*n* = 2), and *garimpeira* (gold miner) (*n* = 1).

Two women reported psychological pressure from clients to engage sex intercourse or unwanted sexual acts, and three reported physical violence for the same purpose (one reported both).

These four cases were not initially captured in the initial question on sexual assault for three of them.

### 3.4 Obstetric history

Twenty-seven women were aged 50 years or older (22.9%) and thus no longer of reproductive age.

#### 3.4.1 Pregnancy

A total of 95/115 (82.6%) women had experienced at least one pregnancy (median: 2; IQR: 1–3.5; maximum: 8). Among them, 93 had at least one child (median: 2; IQR: 1–3; maximum: 7). The median age at first pregnancy was 18 years, and 46.3% reported a first pregnancy before age 18; the youngest age reported was 11 years (Figure 2). There was no statistical correlation between having a pregnancy before the age of 18 and the level of education, country of birth or time in gold mining activity.

**Figure 2:**
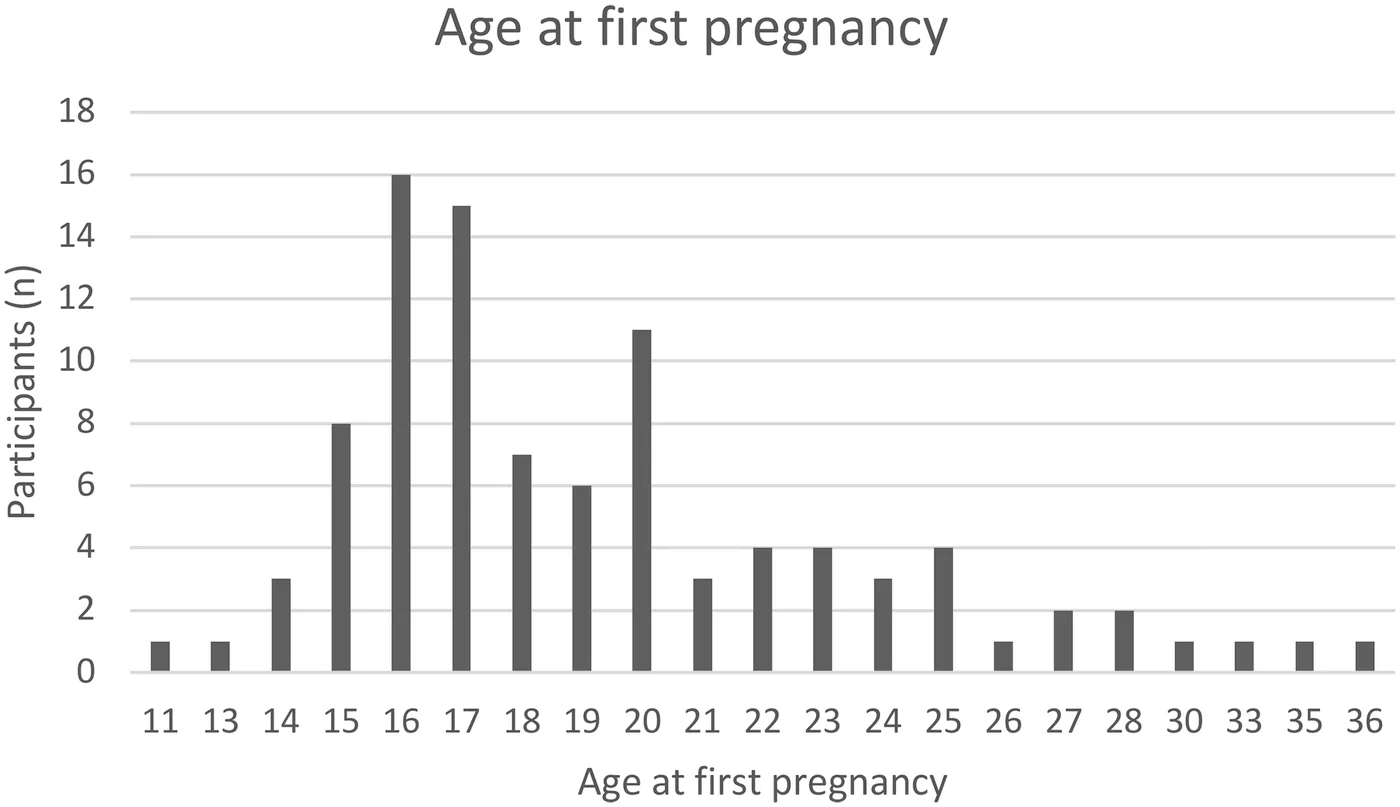
Age at first pregnancy among women involved in gold mining activity in French Guiana, 2024–2025.

The median age at first pregnancy did not differ between women aged ≤49 years and those older than 49 at the time of the study (18 [IQR: 16–22] vs. 19 [IQR: 16–20], *p* = 0.967).

Twenty-three women reported becoming pregnant while working in gold-mining activities (two declined to answer). None were pregnant at the time of the interview. Among these 23 pregnancies, there was one induced abortion and four miscarriages (4/23, 17.4%). Eighteen pregnancies resulted in live births: 11 in Brazil, one in Suriname, and six in French Guiana (three in Cayenne and three in Saint-Laurent-du-Maroni). Seven deliveries were by cesarean section and 11 vaginal, with no difference by country of delivery (*p* = 0.633).

#### 3.4.2 Induced abortion

A third of the women (38/115, 33.0%) knew someone who had had an abortion, most of which were performed by a healthcare provider (25/32, 78.1%). Among these, six participants subsequently referred to a spontaneous or “natural” abortion.

Thirty women reported having had at least one abortion themselves (30/115, 26.1%; 95% CI: 18.3–35.1). Twelve of them then mentioned a spontaneous or “natural” abortion. Therefore, an estimated 18/115 women (15.6%; 95% CI: 9.5–23.6) had experienced an induced abortion. More than half of these induced abortions were performed by a healthcare provider (10/18, 55.6%). When performed at home, a medication was always used, with misoprostol (Cytotec) mentioned by three participants. The places of abortion are described in Figure 3. Abortions were most commonly reported as having been performed by healthcare professionals in Brazil, followed by Suriname and French Guiana, while some occurred at home or in unspecified locations.

**Figure 3:**
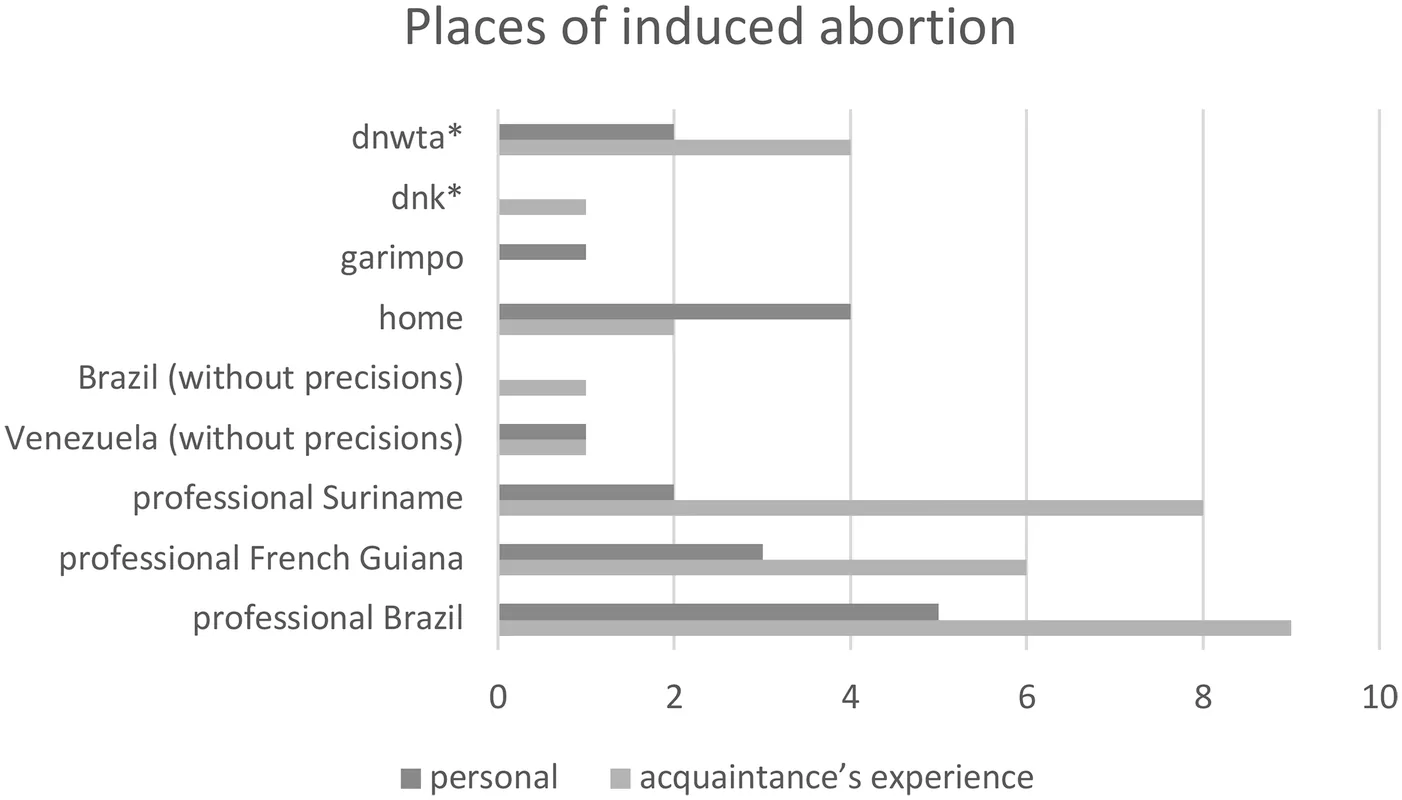
Place of induced abortion for women involved in gold mining activity in French Guiana, 2024–2025. *dnk, does not know/dnwta, does not want to answer.

Reporting having had an abortion was not statistically associated with age (29.2% among women ≤49 years vs. 15.4% among those >49 years, *p* = 0.158), nor with the country of inclusion (Suriname 29.6% vs. Brazil 14.8%, *p* = 0.098), nor with practicing transactional sex (27.7% vs. 33.3%, *p* = 0.419) or education level (low education 34.0% vs. high education 20.0%, *p* = 0.090).

#### 3.4.3 Children

The median age of the youngest child was 8 years (IQR: 3–8; range: 1–39). All children were reported to be in good health. Four were adults living independently; six lived with their mother, five with another family member, one with the father, one with a friend, and one with a paid caretaker. Children living with their mother were aged 1, 2, 4, 9, and 11 years.

### 3.5 Gynecological follow-up and contraception

Most participants (92.2%, 106/115) had undergone gynecological examination at least once in their lifetime, and 92/106 (86.8%) had done so within the last 3 years. Thus, 92/115 women were up to date with gynecological follow-up (80.0%, 95% CI: 71.5–86.8), with no association with age or education.

Half of the women (64/115, 55.6%) reported using contraception, with significant differences by age: 68.5% among women ≤49 years vs. 11.5% among those >49 years (*p* < 0.001). Contraceptive methods are presented in Table 3. Reasons for non-ongoing contraception included menopause (*n* = 31), desire for pregnancy (*n* = 3), absence of a partner (*n* = 1), lack of access to contraception (*n* = 1), or unspecified reasons.

**Table 3:** Contraceptive methods of women involved in gold mining activity in French Guiana, 2024–2025.

Contraceptive method	≤49 Years	>49 Years	Total
Condom	19 (31.2)	1 (33.3)	20
Contraceptive implant	4 (6.6)	0	4
Injection	9 (14.8)	0	9
Tubal ligation—hysterectomy	16 (26.2)	2 (66.7)	18
Contraceptive pill	13 (21.3)	0	13

### 3.6 STIs testing

Among the 115 participants, 75 women (65.2%) reported having previously undergone HIV testing, and 11 (9.6%) were unsure. Among the 86 women who reported or were unsure about having been tested, one did not recall the test result, one reported that the result was still pending, and 84 reported a negative result (97.7%). HIV testing had been performed after 2020 in 92% of cases, and 60% of tests were conducted after January 1, 2024, corresponding to the year preceding study inclusion.

Regarding hepatitis B, 59 women (51.3%) reported having been vaccinated, while 11 (9.6%) were unsure of their vaccination status. In addition, 39 participants (33.9%) reported having previously undergone hepatitis B testing, of whom one reported a positive result, although the specific marker (e.g., HBsAg or serology) could not be specified. Eighty-nine percent of hepatitis B tests were performed after 2020.

Concerning hepatitis C, 33 women (28.7%) reported having been tested, and 9 (7.8%) were unsure. Among those tested, one participant reported a positive result, and one did not recall the result. Ninety percent of hepatitis C tests were conducted after 2020.

Regarding syphilis, 41 participants (35.6%) reported having undergone testing, while 12 (10.4%) were unsure. Among those tested, 10 women (18.9%) reported a positive result, and one did not recall the test outcome. Eighty percent of syphilis tests were performed after 2020.

Twenty-four women reported having been screened for all four STIs. No significant differences in screening uptake was found according to age, education, contraceptive use, up to date gynecological follow-up, history of induced abortion, or prior pregnancy.

Finally, self-reported histories of other sexually transmitted infections were uncommon and included human papillomavirus infection (*n* = 2), gonorrhea (*n* = 1), vulvovaginal candidiasis (*n* = 1), and one report of “piolu”, likely referring to pubic lice infestation.

## 4 Discussion

This study is the first quantitative study to explore sexual and reproductive health among this hidden and hard-to-reach population. One of six women (14.0%) reported sexual assault or rape) and 19.1% reported IPV, with approximately half of these events occurring in a context of alcohol use. One in ten women were currently engaged in sex work, and 8.7% reported past involvement. Abortion concerned 15.6% of women and approximately half of these procedures were performed by a healthcare provider. The first pregnancy occurred before 18 years for half of the participants. Most participants (80.0%) reported up-to-date gynecological follow-up. Among women younger than 49 years, 68.5% reported current contraceptive use. Previous HIV testing was relatively common (65.2%), whereas testing for hepatitis B (33.9%), hepatitis C (28.7%), and syphilis (35.6%) was less frequent.

### 4.1 Limitations and biases

This study has several limitations. The sample size was limited, and the study was not powered to detect associations for sexual and reproductive health outcomes. As a result, analyses were restricted to descriptive statistics, and confidence intervals are reported to aid interpretation. The small sample size and the specific context of illegal gold mining sites limit the generalizability of our findings to other populations and settings.

Given the sensitivity of the topics addressed, particular attention was paid to ensuring confidentiality. Interviews were conducted in a separate, shielded area, and Portuguese-speaking healthcare professionals were recruited, as this is the primary language of most participants. The high acceptance rate—with all women agreeing to respond to this section—suggests good overall acceptability. However, the underreporting of sensitive experiences such as violence, transactional sex or induced abortion is likely, and may have resulted in an underestimation of their prevalence Recruitment took place in busy logistical bases, and partners were sometimes present nearby, creating an atmosphere that may have discouraged some women from disclosing certain events. We also observed that responses to two similar questions asked at different time points and by different interviewers (a physician vs. a community mediator at the Surinamese border) were sometimes discordant (*n* = 3). This may reflect differing levels of trust depending on the interviewer (from the same community or not), or variations in the interpretation of what constitutes violence or victimization (c.f. below).

Another limitation concerns a coding error that restricted questions on antenatal care to women who were pregnant at the time of the survey. As none of the participants were pregnant at inclusion, no data were collected on antenatal follow-up for pregnancies occurring on goldmining sites.

### 4.2 High exposure to sexual and intimate partner violence in mining contexts

In our study, 14.0% of women reported having experienced sexual assault or rape, and 19.1% reported IPV estimates that are slightly higher than global averages, as approximately 11% of women aged 15 years or older worldwide had been subjected to physical or sexual violence by an intimate partner in 2015 (2). These findings are consistent with global estimates indicating that lifetime rape victimization by husbands or boyfriends ranges widely, from 6% to 59%, depending on context and methodology (15). Importantly, these figures likely underestimate the true prevalence of violence, as illustrated by discrepancies between initial negative responses and later mention of coercion or forced sexual acts, highlighting challenges in self-identification as a victim in contexts where violence may be normalized or minimized.

Our results align with qualitative research conducted at the French Guiana–Brazil border, which identifies physical and symbolic violence—particularly intimate partner violence—as a transversal element of women's life trajectories, occurring both before and after migration to mining areas (5). In our study, alcohol consumption was reported in approximately half of the IPV episodes, consistent with previous evidence indicating an association between alcohol consumption and violence against women. A 2019 assessment reported excessive alcohol consumption among 57.3% of individuals working in illegal gold-mining areas, as well as an association with a higher number of sexual partners (7). These findings suggest that alcohol consumption may be part of a broader context of vulnerability involving both violence and sexual health risks. The role of other factors, such as illicit drug use, should be assessed in future studies.

Similar patterns are observed in the broader regional context. In French Guiana, nearly 30% of women report not having consented to their first sexual intercourse (16), while in Brazil, a 2019 national survey found that 19.4% of women had experienced violence at least once in their lifetime, and 1% reported sexual violence in the previous year, often involving threats or physical force (17). The consequences of sexual violence are substantial, with psychological, physical, and sexual health impacts reported by 60.2%, 19.4%, and 5.0% of victims, respectively (17). The women included in our study originated predominantly from Maranhão, Pará, and Amapá, states characterized by low socioeconomic indicators and substantial levels of violence thereby encompassing populations experiencing marked social vulnerability (4, 17–19).

One might have expected higher rates in this context, given that migrant women are often highly exposed to gender-based violence, and given the specific setting of illegal gold mining (20). Indeed, women living on illegal gold mining sites in French Guiana are frequently geographically isolated, with limited access to police or judicial authorities, while the illegal status of mining fosters fear of law enforcement and reluctance to seek help, contributing to structural impunity. Although informal systems of regulation and protection may exist within mining camps, these mechanisms remain poorly documented and may reproduce existing gendered power imbalances (21). As described by Mendes et al., stigmatization permeates women's daily lives in mining environments, where a sexual division of labor embedded in a male-dominated hierarchy devalues women's work, reinforces economic dependency, and heightens vulnerability to exploitation and violence. In addition, some participants spontaneously reported experiences of sexual violence perpetrated by law enforcement personnel during military operations against illegal gold mining, although no quantitative data are currently available to substantiate these allegations. Finally, evidence from industrial mining settings indicates that gender-based violence is not confined to informal or illegal mines, with external reviews documenting pervasive sexism, sexual harassment and assault, and persistent structural inequities across the mining sector (22, 23).

Interprofessional, health system–based centres have been developed to respond to IPV and sexual violence, providing survivor-centred care alongside integrated social, legal, police, and shelter services within a single setting (24). The inauguration of the Maison des Femmes in September 2025 in Oiapoque, a border town between French Guiana and Brazil, represents a timely and promising initiative, illustrating the growing dynamics of cross-border cooperation while supporting women and their children affected by violence (25).

### 4.3 Transactional sex: challenging commonly held stereotypes

The proportion of women reporting current or past involvment in sex work in this study (approximately 10%) is consistent with findings from other studies in gold mines in French Guiana suggesting a stable pattern across contexts (4, 7). This proportion was lower than that reported in mining areas in Mali, where 26.7% of women reported sex work as their main activity (26). However, this estimate should be interpreted with caution. In our questionnaire, sex work was assessed on the basis of women's self-identification, and participants were not asked to provide details about the nature of the exchange involved. Consequently, our measure may not have captured all forms of transactional sexual practices, particularly informal or occasional exchanges of sexual relations for transportation, food, gifts, protection, accommodation, or other basic necessities. Such practices may be normalized and may not be perceived or reported as sex work, especially when no direct payment in cash or gold is involved (5, 27, 28). The prevalence of transactional sexual practices may therefore be higher than the proportion of women who self-identified as having engaged in sex work.

In our study, most women stated that they do sex work indicated working independently, which may indicate the absence of formal organizational control. However, this should not be interpreted as evidence of autonomy or the absence of coercion as it is based on self-reported data and on a relatively small sample size. Mining areas may offer opportunities for some women to sell sexual services (29). These settings, characterized by informality, isolation, and male-dominated power structures, may increase women's vulnerability to violence, coercion, and exploitation even when sex work is initially entered voluntarily. Moreover, human trafficking for sex work is well documented in connection with gold mining, particularly in Colombia and Peru (30).

Condom use was reported to be high in transactional contexts, in line with previous studies showing greater protection with clients than with intimate partners, highlighting persistent power imbalances and limited negotiating capacity within affective relationships (7, 31). In Malian mines, qualitative findings confirm that poverty and boyfriends' refusal to use condoms remain key barriers to systematic condom use among women involved in sex work (26).

### 4.4 High abortion rates despite good access to gynecological care and contraception

In our study, one in six participants reported having experienced an induced abortion. This proportion is likely underestimated given the restrictive legal context in Brazil, where abortion remains criminalized except when the pregnant person's life is at risk, in cases of rape, or fetal anencephaly (32). A recent bill (PL 1,904/2024) sought to further tighten the law by equating abortions performed after 22 weeks with homicide, even in legally permitted situations (33). In such contexts, women frequently conceal induced abortions or report them as miscarriages, leading to systematic underestimation (34). In our sample, several participants initially reported having had an abortion but subsequently described spontaneous miscarriage, which may reflect the stigma surrounding induced abortion. If these 12 ambiguous cases are considered, the proportion may reach one in four women.

Some participants reported accessing abortion through health professionals in Brazil or Suriname, where abortion is prohibited under all circumstances (35). Healthcare professionals may therefore provide abortion care outside the legally authorized framework. The risks associated with clandestine or extralegal practices are well documented, including those related to misoprostol use, which is frequently diverted for abortion purposes in Brazil (36, 37). Resistance to abortion care among healthcare professionals was not reported in our survey as a barrier to accessing abortion services.

Our findings are consistent with Brazilian national surveys reporting a lifetime abortion prevalence of 13%, reaching 15%–18% in the North and Northeast regions, where most participants originated (38). In France—where abortion has been legal since 1975 and is currently permitted up to 14 weeks—abortion rates remain particularly high in French Guiana, reaching 46.7 per 1,000 women aged 15–49 in 2023 compare to 16.3 per 10,000 in France (39). This figure is likely underestimated, as it includes only hospital-based procedures. These indicators cannot be directly compared with our estimate of lifetime abortion prevalence, as French data refer to annual abortion rates rather than the proportion of women who have ever had an abortion.

These high abortion rates persist despite easy access to contraception in France and relatively widespread contraceptive use in Brazil. In Brazil, approximately 62% of women aged 18–49 use contraception, with two-thirds paying out-of-pocket and one-third relying on public healthcare services (40). This proportion appears higher than in French Guiana, where 68% of women not wishing to become pregnant reported not using an effective contraceptive method other than condoms (41).

Although contraceptive knowledge seems relatively good, structural and perceptual barriers remain significant. Difficulties in scheduling appointments, limited availability of methods, and insufficient information may contribute to unintended pregnancies (42). Negative perceptions also play a role, as 43% of respondents believe that oral contraception can cause infertility (16). Improving access to and information on reproductive planning therefore remains a public health priority in both Brazil and French Guiana, particularly in this gold mining population, where half of first pregnancies occur before age 18, consistent with data from Brazil (e.g., 58% of pregnancies occur among 10–19-year-olds in the northern regions) (43, 44). Early pregnancy may have important social and health consequences, including interrupted education, reduced economic opportunities, early union formation, and increased vulnerability to gender-based violence. However, these consequences were not directly assessed in our study (45).

### 4.5 Insufficient screening for STIs

STI screening remains insufficient in this isolated population, which is exposed to gender-based violence and, in some cases, transactional sex. This is particularly concerning given that, globally, women bear a greater burden of sexually transmitted infections in terms of health impact, despite lower incidence rates compared with men (46). Among the general population of gold miners, the prevalence of HIV, HCV, and hepatitis B surface antigen (HBsAg) was 0.5% (95% CI: 0.1–2.1), 2.1% (95% CI: 0.7–3.6), and 1.6% (95% CI: 0.3–2.8), respectively (7). Among the population living along the Maroni River, the corresponding prevalences were 0.65% (95% CI: 0.00–1.40), 0.13% (95% CI: 0.00–0.27), and 2.08% (95% CI: 1.49–2.66), respectively (47). Although these prevalences do not appear substantially higher than those reported in the general population of French Guiana, they nevertheless highlight the need to strengthen STI screening and prevention strategies in this hard-to-reach population (48).

Among women engaged in gold mining in French Guiana, syphilis prevalence is higher than in the general population (7, 49). Women also appear to be more frequently tested than men: in 2018, 82.3% of women working in gold mining sites in French Guiana had previously undergone HIV testing, compared with 55.6% of men (4). In western French Guiana, higher proportions of chlamydia and gonorrhea infections have also been reported among women compared with men (50). Unpublished serological data further suggest a high prevalence of chlamydia among women working in gold mining areas.

A better understanding of barriers to HIV testing is therefore essential. In a qualitative study of 15 women living with HIV, late diagnosis was associated with structural constraints related to illegal gold mining activities (military repression and remoteness from healthcare facilities), health perceptions shaped by precarious living conditions, prioritization of acute health needs and self-medication, and gender norms influencing risk perception and delaying testing (3).

The requirement for two visits (sampling and result disclosure) may hinder testing uptake in this highly mobile population, for whom attending follow-up appointments is often difficult. Some individuals may also assume that any blood test automatically includes STIs screening. Notably, around 10% of women reported not knowing or not recalling whether they had ever been tested. In our study, unfortunately, rapid tests could not be performed, for both practical and regulatory reasons. STIs screening was therefore carried out on sera, and women had to attend French health centers to obtain their results, which may have further limited result retrieval. Rapid diagnostic tests represent a valuable and practical tool in remote settings and should be more widely implemented. Training healthcare providers to systematically offer rapid testing to individuals working in gold mining sites—and more broadly in similar vulnerable populations—would be highly relevant.

## 5 Conclusions

This first quantitative study of a hidden and hard-to-reach population reveals a high burden of sexual violence, early and unintended pregnancies, and under-screened sexually transmitted infections, despite relatively good engagement with reproductive health services.

As with all forms of gender-based violence, effective prevention in mining contexts requires moving beyond victim-centered responses toward structural approaches that improve access to health and support services, reduce harmful alcohol use, and critically engage men to challenge gender norms that legitimize domination, coercion, and violence, without which sustainable impact is unlikely. Given the vulnerabilities faced by women in this region, it is essential that health professionals across the Guiana Shield countries are informed about the challenges encountered by these populations. Systematic screening for sexually transmitted infections, alongside the identification of experiences of violence and potential indicators of human trafficking, should be integrated into routine healthcare encounters when appropriate.

## Data Availability

The datasets presented in this article and the datasets generated during the current study are not readily available because the study population is particularly vulnerable and unrestricted data sharing could pose risks to participants' privacy and safety. However, de-identified data may be made available from the corresponding author upon reasonable request and subject to controlled access, ethical approval where applicable, and appropriate data-use agreements. Requests to access the datasets should be directed to Maylis Douine, maylis.douine@chu-guyane.fr.
