# Supplementary material for: Sexual and reproductive health of women living on illegal gold mining sites in French Guiana: a multicentric cross-sectional survey
Source: Front Glob Womens Health. 2026 Oct 26;7:1887897. doi: 10.3389/fgwh.2026.1887897 (PMC13745568; doi:10.3389/fgwh.2026.1887897)
Supplement: Supplementary file 1 [file Supplementaryfile1.docx]

# Supplementary Material 1

# Survey questionnaire

## Study Title

Radical Cure for Malaria Among Highly Mobile and Hard-to-Reach Populations in the Guiana Shield
Post‑Intervention Survey

**Short Title**

ORPAL_4

# Eligibility Criteria

Do you work in gold mining in French Guiana?
• Yes
• No

Did you leave the mining site less than 7 days ago?
• Yes
• No

Are you over 18 years old?
• Yes
• No

Is this your first participation in the ORPAL_4 study?
• Yes
• No

Signed written informed consent:
• Yes
• No

Inclusion date: … / … / …
Initials of the investigator: …
Inclusion site: …
Participant number: …

# Sociodemographic Data

Gender:
• Female
• Male
• Other (specify): …

If female: Are you pregnant?
• Yes – How many months? …
• No
• Prefers not to answer
• Does not know

Age (in years): …

Country of birth:
• Brazil
• French Guiana
• Suriname
• Guyana
• Dominican Republic
• Other country (specify): …
• Prefers not to answer
• Does not know

If born in Brazil: State of birth:
• Amapá
• Pará
• Roraima
• Maranhão
• Other state (specify): …
• Prefers not to answer
• Does not know

Level of schooling:
• None
• Primary education (1–4 years of school completed)
• Secondary education (5–12 years completed)
• Higher education (university, college, etc.)
• Prefers not to answer
• Does not know

# Gold Mining Activity Data

How long have you worked in gold mining in total?
Indicate duration:
• … days
• … weeks
• … months
• … years
• Prefers not to answer
• Does not know

What is the distance from this mining site, and how long did your trip take to get here (relative to the inclusion site)?
• … hours
• … days
• Prefers not to answer
• Does not know

What is your current occupation at the mining site?
(Multiple answers allowed)
• Chainsaw operator
• Machine operator
• Sex worker
• Boatman / canoe operator
• Transporter / carrier / fuel carrier
• Quad bike driver
• Bar or cabaret worker
• Machine owner
• Mining site manager
• Bar or cabaret owner
• Shop or sales worker
• Shop owner
• Cleaner / cook
• Street vendor
• Prospector
• “Piupiuzeiro”
• Miner
• Unemployed
• Other (specify): …
• Prefers not to answer
• Does not know

Have you experienced a police operation at your mining site during the past year?
• Yes
• No
• Prefers not to answer
• Does not know

If yes:

 How many times?
 • Free text: …

 When was the most recent one?
 • … days
 • … weeks
 • … months
 • … years

# Violence Exposure

Do you know someone (or yourself) who has been a victim of violence?
• Yes
• No
• Prefers not to answer

If yes:

 What type of violence?
 (Multiple answers allowed)
 • Murder
 • Sexual violence
 • Physical violence
 • Psychological violence
 • Other (specify): …

 Where did the violence occur?
 (Multiple answers allowed)
 • Mining site in Brazil
 • Mining site in French Guiana
 • Mining site in Suriname
 • Mining site elsewhere
 • In Brazil
 • In French Guiana
 • In Suriname
 • In another place: …

We are now going to ask you some more personal questions. You do not have to answer if you do not want to.

Do you know anyone who has been the victim of sexual assault, rape, or attempted rape?
• No
• Yes
• Prefer not to answer

 In which country?
 • French Guiana
 • Suriname
 • Brazil
 • Other: …
 • Prefer not to answer
 • Don’t know

Have you ever been the victim of sexual assault, rape, or attempted rape?
• No
• Yes
• Prefer not to answer

 In which country?
 • French Guiana
 • Suriname
 • Brazil
 • Other: …
 • Prefer not to answer
 • Don’t know

 In what year?
 • Free text: …

Has your partner or former partner(s) ever been violent toward you?
• No
• Yes
• Prefer not to answer
• Don’t know

 Did this happen in the context of heavy alcohol use?
 • No
 • Yes
 • Prefer not to answer
 • Don’t know

# Sex Work

Have you ever worked as a sex worker?
• Never
• Occasionally
• Frequently
• I have done it, but I no longer do
• Prefer not to answer

 How many clients did you have in the past year as a sex worker?
 • Free text: …
 • Prefer not to answer
 • Don’t know

 How often do you use condoms with your clients?
 • Never
 • Occasionally
 • Frequently
 • Always
 • Prefer not to answer
 • Don’t know

 In general, where do most of your paid sexual encounters take place?
 • At a gold mining site
 • Elsewhere
 • Prefer not to answer
 • Don’t know

 Is or has condom use been a subject of negotiation with your clients?
 (For example, clients willing to pay more for sex without a condom)
 • Never
 • Occasionally
 • Frequently
 • Prefer not to answer
 • Don’t know

 Has any client ever threatened you or exerted psychological pressure to have sex with you, or forced you to perform sexual acts that you refused?
 • Never
 • It happened in the past 12 months
 • It happened more than 12 months ago
 • Prefer not to answer
 • Don’t know

 Has any client ever used physical force to have sex with you or forced you to perform sexual acts that you refused?
 • Never
 • It happened in the past 12 months
 • It happened more than 12 months ago
 • Prefer not to answer
 • Don’t know

 How is your commercial sex work organised?
 • You are self-employed and work alone
 • You are self-employed, but you pay other people for your security and/or travel arrangements
 • You work for someone else
 • You work in a community and share costs and income with other people
 • Other: …
 • Prefer not to answer
 • Don’t know

# Gynaecological and Obstetric Follow-up – History

How many pregnancies have you had?
• Free text: …

How many children have you had?
• Free text: …

At what age did you become pregnant for the first time?
• Free text: …

Have you ever had a gynaecological examination?
• No
• Yes
• Prefer not to answer
• Don’t know

 How long has it been since your last gynaecological examination?
 • … days
 • … weeks
 • … months
 • … years

Do you use any contraceptive or other methods to avoid pregnancy?
• Yes
• No
• Prefer not to answer

 Which ones?
 • Condom
 • Pill
 • Morning-after pill
 • Intrauterine device (copper or hormonal)
 • Progestogen injection
 • Implant
 • Patch
 • Spermicidal cream, ovule, sponge
 • Ring (Nuvaring®)
 • Withdrawal method
 • Tubal ligation
 • Partner’s vasectomy
 • Avoiding penetration
 • Natural methods (cycle counting, temperature)
 • Other: …

 Where did you obtain it the last time?
 • Health centre or hospital in French Guiana
 • Health facility in Brazil
 • Health facility in Suriname
 • Bought it yourself
 • Other: …

 If you do not use contraception, why?
 • Don’t know
 • Wish to become pregnant
 • Difficult access to contraceptives
 • Menopause
 • Other: …

Do you know anyone who has ever had an induced abortion?
• Yes
• No
• Prefer not to answer

 Where did she have the abortion?
 • Health facility in French Guiana
 • Health facility in Brazil
 • Health facility in Suriname
 • At home
 • Other: …
 • Prefer not to answer

Have you ever had an induced abortion?
• No
• Yes

 How many times?
 • Free text: …
 • Prefer not to answer

 Where did you have the abortion?
 • Health facility in French Guiana
 • Health facility in Brazil
 • Health facility in Suriname
 • At home
 • Other: …
 • Prefer not to answer

# Pregnancy Follow-up

*(if you have had at least one pregnancy and at least one child)*

Have you ever been pregnant since you started working in gold mining?
• No
• Yes, I am currently pregnant
• Yes, in the past

 What was the date of delivery for your last pregnancy?
 • _ _ / _ _ / _
 • Prefer not to answer

The last time you were pregnant, was the pregnancy carried to term?
• Yes
• No, I had an induced abortion
• No, spontaneous abortion
• No, premature delivery
• Prefer not to answer

If premature delivery or carried to term:

 In which country did you give birth?
 • Brazil
 • French Guiana
 • Suriname
 • Guyana
 • Dominican Republic
 • Other: …
 • Prefer not to answer
 • Don’t know

 In which city did you give birth?
 • Free text: …

 How did you give birth?
 • Vaginal delivery
 • Cesarean section
 • Prefer not to answer

 How old is your youngest child?
 • … days
 • … weeks
 • … months
 • … years

 How is he/she doing?
 • Fine
 • Other: …
 • Prefer not to answer
 • Don’t know

Who takes care of your children?
• The participant herself
• With the father
• With the participant’s family
• With friends
• With a paid caregiver
• Living alone as an adult
• Other: …
• Prefer not to answer
• Don’t know

 Where are they?
 • French Guiana
 • Suriname
 • Brazil
 • Other: …
 • Prefer not to answer
 • Don’t know

 Do you take your children to the gold mining site?
 • Never
 • Occasionally
 • Frequently
 • Not applicable
 • Prefer not to answer

# STIs’ Screening

Have you ever had an HIV diagnostic test?
• No
• Yes

 Year: …

 Result:
 • Positive
 • Negative
 • I don’t remember
 • Prefer not to answer
 • Don’t know

Have you been vaccinated against hepatitis B?
• No
• Yes, I am sure
• I think so, but I am not sure
• Prefer not to answer
• Don’t know

Have you ever had a hepatitis B diagnostic test?
• No
• Yes

 Year: …

 Result:
 • Positive
 • Negative
 • I don’t remember
 • Prefer not to answer
 • Don’t know

Have you ever had a hepatitis C diagnostic test?
• No
• Yes

 Year: …

 Result:
 • Positive
 • Negative
 • I don’t remember
 • Prefer not to answer
 • Don’t know

Have you ever had a syphilis diagnostic test?
• No
• Yes

 Year: …

 Result:
 • Positive
 • Negative
 • I don’t remember
 • Prefer not to answer
 • Don’t know

Have you ever had a sexually transmitted infection?
• No
• Yes, confirmed and treated
• I think so
• Prefer not to answer
• Don’t know

If yes, confirmed and treated:

 Which one(s)?
 • Gonococcus
 • Chlamydia
 • Urethritis
 • Condyloma
 • Human papillomavirus (HPV)
 • Other: …
 • Prefer not to answer
 • Don’t know
